# Economic value of protected areas via visitor mental health

**DOI:** 10.1038/s41467-019-12631-6

**Published:** 2019-11-12

**Authors:** Ralf Buckley, Paula Brough, Leah Hague, Alienor Chauvenet, Chris Fleming, Elisha Roche, Ernesta Sofija, Neil Harris

**Affiliations:** Griffith University, Gold Coast, QLD Australia 4222

**Keywords:** Environmental economics, Economics

## Abstract

We evaluate methods to calculate the economic value of protected areas derived from the improved mental health of visitors. A conservative global estimate using quality-adjusted life years, a standard measure in health economics, is US$6 trillion p.a. This is an order of magnitude greater than the global value of protected area tourism, and two to three orders greater than global aggregate protected area management agency budgets. Future research should: refine this estimate using more precise methods; consider interactions between health and conservation policies and budgets at national scales; and examine links between personalities and protected area experiences at individual scale.

## Abstract (web summary)

Parks have a previously unquantified economic value attributable to mental health, a health services value. Here, the authors proposed three methods to estimate this, and applied one of these methods to show that this value is at least US$6 trillion per annum worldwide.

## Introduction and conceptual framework

Conservation is key to sustainability^1–3^, but biodiversity continues to decrease worldwide^4–6^. Protected areas remain the core of global conservation strategies^7,8^, but are under increasing pressure^9–11^ from political and economic factors^12,13^ as well as climate change^14^. Conservation relies on political advocacy, influenced by economic arguments^2,15^, based on ecosystem services^16^ or tourism^17^. Nature exposure improves human mental health and wellbeing^18–21^. Poor mental health imposes major costs on human economies^22–24^. Therefore, parks have an additional economic value through the mental health of visitors^25^. We refer to this as a health services value. This may be considered as a component of ecosystem services value^26^. Here we consider how to calculate health services value.

Research on nature exposure and mental health falls into four main categories^20^: spatial correlations between nature access and mental health^27–29^; joint patterns across populations^30,31^; experimental tests linking nature exposure to specific psychological parameters^18–22,32–34^; and qualitative analyses examining the psychological processes underlying these links^35,36^. Health-related benefits include improved attention^32^, cognition^33^, sleep^37^, and stress recovery^38^, and apply across demographic and socioeconomic population sectors^18,19,25^. Research on economic costs of poor mental health recognises four main categories: treatments^39,40^, both consultations and pharmaceuticals; caregivers, both paid and unpaid (e.g. family members); lost workplace productivity, through absenteeism^39^ or poor performance (presenteeism)^41^; and antisocial behaviour^42–44^, both public (e.g. vandalism) and private (e.g. domestic violence).

Human economies have underinvested severely in nature conservation, despite the high value of ecosystem services^16^, because these services have been provided free of charge. The same applies for health services, but we suggest that there may be one key difference. In agrarian and manufacturing economies, the relationship between individual mental health and society-scale economic performance is a step function: irrelevant, until it is severe enough to generate crime or workplace absenteeism. In professional and service economies, however, the relationship is gradual: poor mental health decreases contributions to employers and society. Powerful stakeholders therefore have financial interests in individual mental health. Health services value thus has new importance in conservation policy. Here, therefore, we estimate its economic scale, relative to ecosystem services, biodiversity prospecting, or tourism.

## Valuation methods

### Methodological approaches

**Fig. 1:**
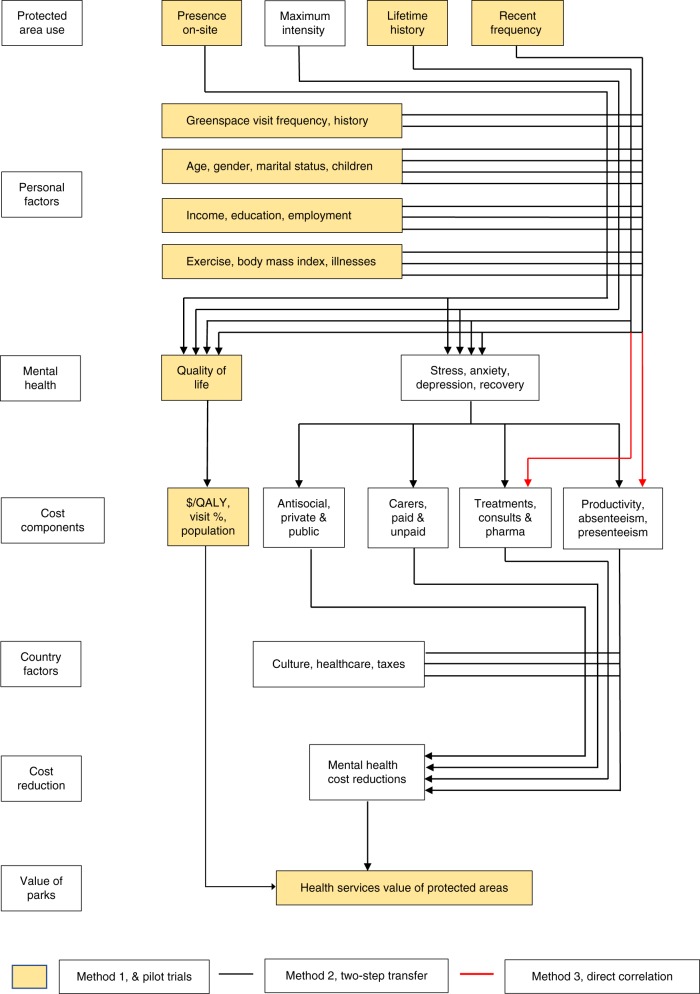
Flowchart of calculation pathways for health services value of protected areas. Seven-block flowchart of calculation pathways for health services value of protected areas, showing three major pathways. History, frequency, and intensity of protected-area visitation (block 1) affects mental health and quality of life of park visitors (block 3), but these are also affected by demographic and socioeconomic factors, physical health factors, and non-park greenspace use (block 2), which must therefore be controlled for. Costs (block 4) can be linked to mental health either via quality-adjusted life years (yellow boxes), individual cost components (black lines), or direct correlation with park visit patterns (red lines). Cost reductions (block 6) also depend on national healthcare funding systems (block 5). The health services value of protected areas (block 7) is the net reduction in aggregate costs of poor mental health, derived from visitation to protected areas. The three pilot studies presented here use the $/QALY pathway, shown in yellow boxes, with three different measures of park use (block 1)

We propose three potential valuation approaches, based respectively on: quality-adjusted life years; two-step transfer functions; and direct correlations with costable parameters (Fig. 1). We examine the theoretical and practical advantages and disadvantages of each, including requirements for additional data. For the first approach, which needs fewest new data, we present three pilot trials. We consider how valuations can be scaled up nationally and globally, and identify priorities for future research

There are three obstacles that apply across all three of these methodological approaches. The first is that the effects of nature exposure depend on type and timescale^18–20,25,45–52^. This leads to uncertainty over how best to define patterns of use for protected areas, and how to differentiate protected areas from non-park greenspace. The second is that mental health may be a driver as well as a consequence of protected area visitation, so separate tests of causation are necessary^36,49,50^. The third is that mental health is affected by many factors other than nature exposure, so extensive controls are required. Such factors include demographic and socioeconomic parameters, and physical health, fitness, and lifestyle^53–57^.

### Quality-adjusted life years

The first approach uses a standard concept in health economic policy, namely quality of life, QOL^58^, and quality-adjusted or disability-adjusted life years, QALY or DALY^53,59^. QALYs are used routinely throughout healthcare design, policy, evaluation, and research.^53,54^ They are used for mental as well as physical health conditions.^60–62^ For example, they have been used for physical exercise in parks^63^, cancer survivors^64^, and mental health therapies^65,66^, though not previously for the mental health services value of protected areas. There are also standard medical measures of QOL^67^, such as the Personal Wellbeing Index, PWI^68^, recorded annually by national statistical agencies. The same concepts and questions are used to measure QOL and QALYs. Therefore, if we can show how park use patterns affect QOL, we can convert to financial figures using economic values per QALY unit, $/QALY.

Numerous meta-analyses have attempted to quantify and standardise $/QALY.^69–75^ These figures have been calculated repeatedly for many countries, using real-life marginal willingness to pay for different treatments for various conditions, and revealed valuations for improved longevity and quality of life. Estimates differ between countries, but not greatly^76–79^. Estimates may also depend on: individual socioeconomic circumstances^80^; assessment for oneself cf. assessment for others^81,82^; age^83^; and specific medical conditions^84,85^; notably cancers^86^. A wide range of philosophical, ethical and political issues have been canvassed^87–90^, and a range of variants and alternatives have been proposed^91–95^, but none have yet been adopted. Numerous recent studies place the value of a QALY in the range US$50,000–250,000^92,96–99^, with slightly higher values from Japan^76^. For most developed nations, where 80% of worldwide park visits occur^17^, $/QALY = US$150,000–250,000.

The QOL/QALY pathway is robust, reliable, and readily scaleable. It is widely used in real-life health policy and economics. It includes individuals at all levels of mental health, since QALY aggregates a near-continuous measure of QOL. $/QALY is calculated specifically from changes in QOL due to changes in health, not other factors. Statistical calculation is robust, a one-step multiple regression. Sources of uncertainty are transparent: possible reverse or reciprocal causation (<18%), which applies across all methods, and variation in mean $/QALY between countries (<20%). The PWI measure of QOL has already been translated and validated in 25 languages and countries.

The QOL/QALY measure yields a more conservative estimate than currently computable alternatives such as the experience preference (EP) method^100^, which trades park visits against income, using a QOL yardstick. EP is a revealed preference method, more reliable than stated-preference willingness-to-pay. EP is numerically precise, but it includes an undefined value for happiness, in addition to mental health. For our data, EP yields a value 60% higher than the QOL/QALY path.

### Two-step transfer functions

The second approach involves a two-step matrix of transfer functions, first from various measures of protected area use to various measures of psychological health, and then from each of those measures to the multiple cost components associated with poor mental health. The principal advantage of this approach is that it matches most closely to the underlying conceptual framework for estimating health services value^25^: nature improves mental health, and improved mental health reduces costs. There are many standard psychological measures of mental health^101–103^, and numerous prior studies showing the various demographic and socioeconomic factors that influence it^45–54^. In theory, new research needs only to add patterns of protected area use as one additional factor.

In practice, however, this pathway faces several obstacles. It involves concatenated calculations using different techniques, with unknown uncertainties. Psychological scales such as GHQ and K10 yield near-continuous variables, but for medical statistics these are converted to dichotomous variables, healthy/clinical, at cutoff thresholds^100–103^. Conversions from psychological scales to costable measures are thus step functions: scores above cutoff thresholds are assumed to incur mental health costs^39–44^. These scales and cutoffs, however, are designed as medical diagnostic tools, not definitions of clinical conditions. Many individuals classified as clinically unhealthy do not seek treatment, and some classified as healthy do seek treatment^104^. Cutoff thresholds differ between countries^105^. Only some individuals who are clinically diagnosed and receive treatment also have carers; only some are in the workforce and are absent or less productive at work; and only some engage in antisocial behaviour. Some cost components are calculated only for clinically diagnosed patients, others for entire workforces or populations.

For precise calculation, we need numerical data on each of these components. Currently, however, statistics on mental health costs are available only as large scale and imprecise per capita means^25,106^. In addition, with current health statistics, the multi-step method only measures economic value for individuals where visits to protected areas convert them from clinically unhealthy to clinically healthy, a detected therapeutic effect. Protected area visits, however, also improve mental health for individuals who remain clinically unhealthy (undetected therapeutic effect), and for those who remain healthy (preventive effect), and these effects also generate economic value. Estimates using this pathway are therefore not yet reliable.

### Direct correlations with costable parameters

The third approach is to examine correlations directly between parameters of park use as above, and costable parameters of mental health: for example, absenteeism at work or visits to mental health practitioners. That is, this third approach is a shortcut version of the two-step transfers, without explicitly incorporating the intermediate mental health component. The advantage of this approach is that we can measure these costable parameters directly, for the same individuals as park use, generating quite precise valuations. It has three main disadvantages. First, there are many different lifestyle, material, health, and social circumstances, additional to socioeconomic, demographic, and mental health measures, that may influence costable parameters such as workplace absenteeism. Examples include workplace dynamics, and family childcare and schooling.

Second, it confounds causal mechanisms at the scale of individuals, and differences in lifestyles between population subsectors. For example, there are differences between those in full-time employment, those in casual employment, and those who are retired or unemployed. Third, it measures only some of the cost components for poor mental health. To include other components, we must assume that they apply in the same ratios as for national-scale statistics on mental health costs, across all levels of park use. In Australia, for example, direct healthcare costs comprise only 5.5% of total economic costs of poor mental health^107–109^. This assumption is untested.

## Pilot studies using QALY

### Park visitors, on-site

We measured QOL for visitors at the principal trailheads of two Australian subtropical national parks, via the Personal Wellbeing Index^68^, administered face-to-face on-site. We compared visitor PWI against national statistics^107–109^, to estimate per capita differentials, ΔPWI. We used published estimates of $/QALY, as reviewed earlier, to convert ΔPWI to $/visitor. To estimate total annual value for Australia, we then multiplied by protected area visitation rates: 54% of Australia’s adult population of ~20 million are reported to visit national parks at least annually^110^.

Our sample was demographically representative, and we found no significant within-sample differences in mean PWI related to gender, age, income, residence or travel distance, so ΔPWI in this small sample was due specifically to national-park visitation. Mean PWI for these protected-area visitors was 81.78 (s.d. 10.58, *n* = 203). This is significantly higher (*p* < 0.0001) than the Australian population mean^109^ of 76.11, with mean differential ΔPWI = 5.67%. Within-sample PWI was influenced by employment status, and population-weighted mean ΔPWI across the four principal employment categories was 4.86%. At park use frequency of 54%^110^, this indicates mean ΔPWI = 2.4% across the entire Australian adult population. At a frequency of 70%, as reported below, ΔPWI = 3.4%.

### Population sample, past year

Using a commercial online survey organisation that maintains population panels, we compiled relevant data for an Australian population sample (*n* = 19,674), representative of population patterns in gender, age, income, and residential location. The sample was drawn 75% from the tropical and subtropical State of Queensland (*n* = 14,601), and 25% from the southern temperate State of Victoria (*n* = 5073). We found no differences between States, and combined the data for analysis.

Data collected included protected-area and non-park greenspace use over the previous 12 months (P12, G12, log-transformed); QOL, measured as PWI^68^; and socioeconomic, demographic, and physical health parameters. These included gender, age, education, employment, income, marital status, number of children, exercise regime, physical health rating, and body-mass index. We did not include broader measures of neighbourhood socioeconomic status or greenspace type used in urban planning approaches^111^. We defined “protected area” as named National Parks, managed by national parks agencies. Natural areas with other tenures, or managed at local government level, were defined as non-park greenspace. We used multiple regression to express individual PWI as a joint function of these parameters. G12, physical health, exercise, income, employment, education, and a partner, all have significant positive effects on PWI. We then compared the differential between PWI including the effects of P12, and PWI with all effects except P12.

**Fig. 2:**
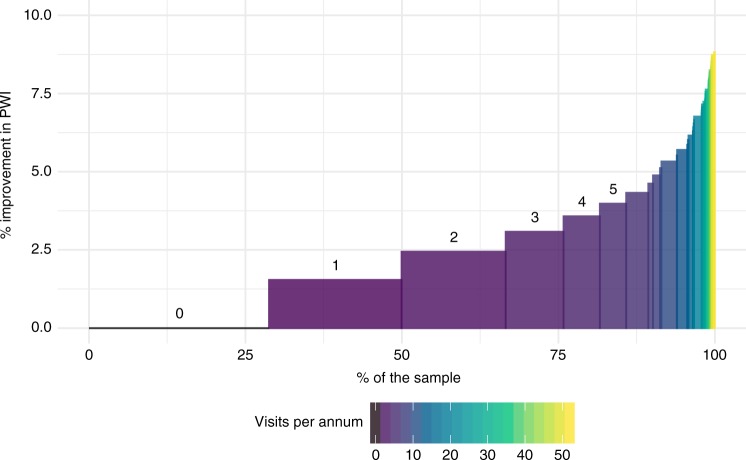
Distribution of quality-of-life improvement derived from protected area visitation. Distribution of quality-of-life (QOL) improvement derived from protected area visitation across the sample population, pilot trial 2. Vertical axis shows QOL improvement, measured as % increase in self-perceived personal wellbeing index, PWI, controlled for socioeconomic and demographic factors and non-park greenspace use. Horizontal axis shows proportions of sample population. Colours show the number of visits to protected areas during the preceding 12 months. For low annual visit frequencies, 0–5 inclusive, frequencies are also indicated by numerals above the bars. Thirty per cent of the sample had not visited parks at all during the past year, and hence experienced no improvement in PWI. The majority of the sample population, shown in purple, had visited a protected area 1–5 times in the preceding year, yielding small but significant improvements in self-perceived wellbeing. Much smaller proportions of the sample population, shown in blue, green, and yellow, had visited monthly, weekly or daily, with improvements in PWI up to ~8%

We found that 30% of the sample population had not visited a named national park during the previous year, and 20% had visited only once. Less than a quarter of the sample had made 4 or more national park visits over the past year. Mean frequency of visits, including those with zero visits, was 2.6 visits per year. Both national parks and non-park greenspace use have separate statistically significant effects on PWI, but the effect size for parks is several times greater than that for greenspace. Across the entire sample population, including those who do not visit protected areas at all, the mean effect of (log-transformed) protected area visitation is ΔPWI = 2.2%, or 3.0% if exercise effects on mental health are excluded (Fig. 2).

### Population sample, lifetime history

We used the same sample as above, plus individual recollected lifetime history of parks and non-park greenspace use. We asked respondents to score as an ordinal variable, average use during each decade of their lives, starting with “childhood” at age 5–15, and continuing to the present. The five ordinal categories ranged from 0 to 1 visits/year, up to >50 visits/year. For more accurate recollection, they were expressed in terms of visits per year, month, or week. Individuals with greater interest in nature may potentially recall higher visit frequencies^112^.

We found that recollected park visit frequency per decade is correlated across decades. Individuals who visited parks either more or less often early in their lives continued to do so throughout later life. The frequency distribution is heavily skewed, with many respondents visiting rarely, and few visiting often. We therefore re-coded lifetime park-visit and greenspace-visit frequencies, LPF and GPF, to the proportion of decades for which respondents recalled visit frequencies above the lowest; i.e., the proportion for which they visited at least twice annually. This also overcomes potential recall bias as above.

Following the same approach as for P12 in the previous pilot study, we found that LPF has a significant effect on PWI even after controlling for socioeconomic, demographic, physical health, and greenspace-visitation variables. The increase in PWI attributable to lifetime protected-area use of at least several times per year averaged across the entire Australian population including non-users, is ΔPWI = 3.1%.

## Scaling up

### Sample to Nation

These three pilot studies each yielded a mean increase in QOL (as ΔPWI) attributable to protected area visitation, across the entire Australian adult population including non-users, controlled for demographic, socioeconomic, and physical-health factors. The first pilot study, for visitors on-site, found ΔPWI = 2.4–3.4%. The proportion of the population visiting national parks at least annually is 54–70%. The second, based on the frequency of protected area visits during the preceding year, found ΔPWI = 2.2–3.0%. Mean frequency across the entire population was 2.6 times/year. The third, based on reported lifetime protected area visitation of at least twice annually, found ΔPWI = 3.1%. Each of these adopts a different way to estimate protected area visitation.

Using the conservative estimate ΔPWI = 2.5%, $/QALY = US$200,000 as above, and the Australian adult population as 20 million, the annual health services value of Australia’s national parks is ~US$100 billion, in addition to values from biodiversity, ecosystem services, and tourism expenditure. This is about 7.5% of Australia’s GDP, 1.6 times the entire annual turnover of Australia’s tourism industry, and two orders of magnitude larger than the aggregate annual budget of Australia’s national parks agencies.

### National to global

To calculate global health services value, the most accurate and reliable approach will be to conduct national-scale representative population surveys in each country worldwide^25^. Nation-by-nation protected-area visitation, mental health statistics, and validated PWI questionnaires are already available for many countries^17,23,113^. Protected area visitation patterns, however, and the dependence of QOL on age, income, education, gender, family factors, and exercise, may differ considerably between countries and cultures^76,114,115^, and these data are not currently available. The proportions of national populations who suffer from diagnosed mental health conditions also differ considerably between nations.

Preliminary estimates of global health services value are available by scaling up from the Australian calculations. Potential scale-up parameters include protected area visitors; protected area visits; GNP; population; or disability-adjusted life years, DALYs, from mental and behavioural disorders. These yield global estimates of US$ 4–31 trillion p.a. (Table 1). Each has shortcomings. Estimates based on DALYs or population do not consider the different values ascribed to $/DALY or $/QALY in countries with different economic circumstances. Estimates based on protected area visits or visitors are effectively biased to developed nations, where 80% of visits occur^17^. Comparing these estimates, a conservative consensus may be around $US6 trillion p.a. This is ~8% of 2017 global GNP of ~US$74 trillion^116^, and ~4% of direct ecosystem services, valued at ~2018US$150 trillion per year^17^. It is 6× the global economic value of outdoor tourism, ~US$1 trillion p.a.^117^, including US$0.6 trillion p.a. from protected area visitation^17^.

**Table 1:** Scale-up from Australian to global health services value

Scaling factor	Unit	Value for Australia	Value for World	Aust as % World	Global US$ trillion
Annual park visits	Billion	0.15–0.20	8	1.9–2.5	4.0–5.3
GNP, US$${\$}$$	Trillion	1.11	74	1.5	6.7
Annual park visitors	Billion	0.011	1.0–1.2	0.9–1.1	9.1–11.1
Mental disabilities^a^	DALY^a^	671	185190	0.36	27.8
Population	Billion	0.024	7.5	0.32	31.3

Data from Australian state and national agencies; global agencies; and research articles^18,23,112–119^

^a^Burden of mental and behavioural disorders, excluding neurological, measured in disability-adjusted life years

## Research priorities

### Triangulation and internationalisation

To date we have only three pilot trials, for only one of three potential valuation methods. The first research priority is thus to triangulate these initial estimates by applying the other two methods. To achieve this, the methodological obstacles outlined earlier must first be overcome. Our pilot trials only include individuals aged >18, but adult and childhood health and wealth are strongly linked^120–122^, so if possible, future studies should also include children. In addition, to date we have valuations only from one country, with global estimates through very approximate upscaling. To generate reliable global figures, we need to repeat at least the QOL/QALY approach, and preferably all three, in multiple countries.

### Causation

The three methodological approaches outlined above all rely on correlations between mental health measures and corresponding cost savings, and patterns in park visitation. The three pilot trials show that protected area users incur and impose lower-than-average mental health costs, but they do not test explicitly for causation. Previous studies using randomised controlled trials, RCTs, did test the causal effects of specific exposures to nature, on specific psychological parameters, for particular groups of individuals^18–20,32–34^. These, however, may not necessarily be transferable to entire national populations, where individuals decide whether or not to visit protected areas, at their own discretion. Therefore, we need to test causation.

There are three main potential approaches. The ideal is a multi-year panel study or lifetime cohort study, simultaneously tracking changing protected area use and mental health for a large, demographically and socioeconomically representative population sample. This is feasible at large scale, but has not yet been attempted. The second is through RCTs. These are difficult to apply in long-term lifestyle comparisons, since individuals would have to visit protected areas (or not) according to a predefined experimental regime, irrespective of their own preferences. Even if they did, conflicts between experimental prescriptions and personal preferences might influence outcomes. This approach is thus feasible only at small scale.

The third is to use social-science approaches, asking individuals about their own perceptions of causation in their own lives. For the current study, we conducted semi-structured interviews with 238 visitors to national parks and nearby greenspace in subtropical Australia. Of those who identified positive emotional outcomes, 82% said that visits make them happy. Of the remainder, some identified a lifetime process of reciprocal feedback between visits and happiness, and others said that happiness depended on factors unrelated to visits.

### Timescale and repetition

Health services value depends on the duration of effects from nature exposure on mental health, as well as the frequency of protected area visits. Just as pharmaceutical treatments are packaged and prescribed to maintain a continuous concentration in the patient’s bloodstream, protected area visits will provide a more effective mental health therapy if the effects of each visit last until the next. If effects are brief and visits infrequent, then mental health gains, and their economic values, will be fractional rather than full-time. There has been little research to date on the decay curve of mental health effects from nature exposure. Different effects operate over different timescales. Powerful emotions can arise and decay within seconds or shorter, but can be remembered for decades^123^. Recovery from stress or anxiety can commence within minutes, but takes hours or days for full effect^124^. Over years and decades, some individuals experience changes in worldview, influencing lifestyle and political beliefs.

Our pilot trials as above, adjusting for the population proportions who do not visit protected areas at all, show little or no decay in ΔPWI from on-site experience to past-year recollection and multi-decadal history. This suggests that QOL effects are quite durable. This matches previous findings^125^ that the psychological effects of nature exposure can persist for at least 3 years. Greater reliability will require panel studies, with resampling over months and years. We also need to consider the potential effect of habituation and withdrawal. For individuals who do experience regular nature exposure, increasing frequency or intensity of exposure may be required to generate the same mental health benefits. This pattern occurs for high-adrenalin outdoor activities^121^, but there are no data as yet for contemplative visits to protected areas. The same applies for withdrawal effects. If a person’s mental wellbeing depends on visits to protected areas, will they suffer greater costs if they lose that opportunity? Research in outdoor tourism may prove applicable, as outlined below.

### Fine-graining: nature components and experiences

Experimental tests of the psychological effects of nature exposure show substantial differences in effects between different types of exposure, different psychological parameters, and different individuals^18–20,45–50,126,127^. Such differences are also likely for visits to protected areas, but as yet unstudied. To identify the effects of protected area visits, we need to know whether mental health effects depend principally on, e.g., time since most recent visit, length of visit, frequency of visits in recent months, lifetime visit history, highly memorable visits, or some other parameter. Since there is no current research on this, we need to compile multiple measures, and compare them empirically to determine which has greatest predictive value for mental health. Ideally, we would measure visitation patterns for cohorts of individuals over years or decades, via a panel study.

Similarly, research on psychological effects of nature exposure has employed a range of experimental interventions, but there have been no systematic tests to compare outcomes from different types or components of real-life visits to protected areas^49,50^. For example, such visits may include scenery provoking awe^128^, wildlife interactions provoking physiological and psychological responses^129–132^, or the enjoyment of breathing unpolluted air. If mental health gains depend on details of experience, as seems likely, that will also affect the health service values of protected areas.

Research in parks and wildlife tourism indicates that such effects are widespread and powerful. Nature-based tourism enterprises devote considerable effort to emotional choreography^129–132^, designing customer experiences that create unanticipated positive emotions, usually referred to as delight. Awe, calm, and joy are also highly valued emotional responses. We therefore need to test firstly, which particular psychological responses create greatest mental health gains; and secondly, when and how such effects can be generated by visiting protected areas. Research in outdoor education^133,134^ has also tested how to improve mental health, with a focus on self-reliance. Economic analyses in nature tourism have calculated values for encounters with individual animals. We could calculate health services value at all scales from population, to insurer and employer, to individual.

### Individual personal characteristics

Equally, there is little information on the effects of differences between individuals. Individual mental health, whether internally or externally perceived, can vary rapidly over time. It can also differ greatly between individuals, depending on temperament and personality as well as life history and material factors. Does the same nature exposure affect everyone equally, or very differently? What do we need to know about individuals, and their protected area visits, in order to calculate health services values? There is ample research on the many non-nature factors affecting mental health^51–54^, and we controlled for these in the pilot trials. The psychological outcomes of nature experiences, however, may also depend on the characteristics of the individuals involved^126,127^. Culture, upbringing, fitness, and familiarity with the outdoors may all influence preferred experiences, from urban to garden, formed park trails to wilderness^114,115^. Just as clinical therapies consider the characteristics and symptoms of individual patients in order to prescribe particular treatments, dosages, and durations, the same may apply for mental health gains through protected area visits^19,49–51^. The mental health value of visiting protected areas differs between visitors, and the health service values of protected areas depends on the visitors as well as the parks.

### Conservation policy considerations

From a policy perspective, conservation is a social machine^135^, an assemblage of interacting components. Economic valuations are one driver, and health service valuations can contribute. Health services value also has political influence, since it links conservation to individual health. Conservation benefits everyone a little, but no individual greatly; individual health benefits individuals greatly, but society only a little. Ecosystem services value connects a global goal to a society-scale interest, whereas health services value connects a global goal directly to individual self-interest, which is more influential. Health services value can therefore increase political as well as economic support for conservation.

Governments, however, distinguish strongly between valuations and cashflows. Health services value will have more influence on national conservation policies if it reduces budget expenditure, or generates taxable revenue. For the former, we need to calculate an RoI: the financial return to government, via reduced health expenditure, on investment in protected areas, via parks agency budgets^136^. This depends on the health policies of individual countries, since these determine what proportions of each of the four major costs of poor mental health^39–44^ are met from taxes. These policies include subsidies and tax rebates for medical consultations, pharmaceuticals, carers, and health insurance premiums, which differ greatly between countries. Even if reallocating budgets from health to conservation portfolios would achieve a positive RoI, private and government healthcare organisations may oppose it, if it lessens their revenues or influence.

Generating new taxable revenue would require routine adoption of outdoor therapies dependent on nature in protected areas^49,50^. As private enterprises and public healthcare agencies perceive mental health opportunities from people visiting protected areas, however, they will try to gain control over access, as an opportunity for profit^50^. This is already a global concern in tourism, where entrepreneurs and industry associations constantly lobby for exclusive controls and development opportunities within public protected areas. There is the same risk for healthcare enterprises, which may combine forces with tourism. Commercial interests in protected area management increase environmental impacts and decrease social equity. Economically disadvantaged individuals in developed nations, and many citizens in developing nations, already face barriers to visiting protected areas. In developing conservation policy to incorporate health services value, therefore, social equity should also be considered.

## Conclusions

We conclude that there is a direct link between protected area visits and individual human mental health and wellbeing, which translates to a very substantial but previously unrecognised economic value for protected areas and conservation. This health services value already exists, since the costs of poor mental health would increase if protected areas ceased to exist, or if people could no longer visit them. Historically, it has not been included in debates over economics and finance for either conservation or health. We argue that it should be recognised, quantified accurately and widely, and included explicitly in policy.

The pilot calculations presented here indicate that health services value is ~4% of global ecosystem services value. This first estimate is imprecise, but that does not matter. What matters is that it is large enough to merit more detailed analysis and application at national and local scales, and potentially, to become a powerful new tool in global conservation. Conservation policy did not change greatly when the global value of ecosystem services was first calculated, and nor will it change solely because of the current calculation. What counts are decisions by individuals controlling financial resources, such as health insurance corporations and government treasuries. They will act once they see immediately implementable options to reduce costs, increase revenue, or buy political power. They may be opposed by interests who stand to lose, such as the pharmaceutical industry^50^.

For governments, the next step is to construct and cost options for public-health portfolios and programs, using budget-estimate and RoI methods adopted by their own Treasuries^136^. In Australia, the aggregate costs of poor mental health currently amount to ~10% of GDP^25^. The pilot estimates presented here indicate that without protected areas, these costs would be 7.5% greater. For protected area management agencies, the key conclusion is that operational management and infrastructure that encourages individual visitors to visit public protected areas contributes substantially more to national economies than arrangements to increase commercial tourism. In addition, if nature therapies become widespread, that may bring more revenue, but also more costs and risks with increased numbers of visitors, some with mental disabilities. Instead of increasing their budgets, governments may expect them to raise revenue through fees and charges. These may introduce equity issues, if individuals who gain most from visiting parks, currently free or cheaply, become unable to afford visits.

For insurers, the next step is to design diagnosable, prescriptible, insurable, and deliverable courses of outdoor nature-based mental health therapy, as a routine component of mainstream medical care^50^. This will require partnerships between general medical practitioners, specialist psychologists, protected area management agencies, and the outdoor education, recreation and tourism sectors, who have the practical skills to deliver such courses^36^.

For researchers, more accurate calculations will require global efforts comparable to those already applied for ecosystem services. This will include large-scale data collection on quantitative links from mental health to cost components; international parallel studies in countries with different cultures, conservation, and healthcare systems; the complexities of individual causation, vs. subpopulation patterns; timescales of nature exposure effects, single or repeated; fine-grained effects of differences in nature experiences and the characteristics of individuals; and incorporation into conservation policy based on returns on investment.
