# Author Correction: Economic value of protected areas via visitor mental health

**DOI:** 10.1038/s41467-019-13619-y

**Published:** 2019-12-10

**Authors:** Ralf Buckley, Paula Brough, Leah Hague, Alienor Chauvenet, Chris Fleming, Elisha Roche, Ernesta Sofija, Neil Harris

**Affiliations:** Griffith University, Gold Coast, QLD Australia 4222

**Keywords:** Environmental economics, Economics

Correction to: *Nature Communications* 10.1038/s41467-019-12631-6, published online 12 November 2019.

The original version of this Article contained an error in the spelling of the author Alienor Chauvenet, which was incorrectly given as Ali Chauvenet.

Reference [90] originally incorrectly read ‘Rutstein, S. E. et al. The annual burden of seasonal influenza in the US veterans affairs population. *Glob. Publ. Health* **12,** 1269–1281 (2017).’ The corrected version of this reference is ‘Rutstein, S. E. et al. Hidden costs: the ethics of cost-effectiveness analyses for health interventions in resource-limited settings. *Global Public Health* **12**, 1269–1281 (2017).’

Reference [91] originally incorrectly read ‘Benson, T. We propose a novel measure for social welfare and public health: capability-adjusted life-years, CALYs. *J. Med. Econ.* **20,** 107–113 (2017).’ The corrected version of this reference is ‘Benson, T. The load model: an alternative to QALY. *J. Med. Econ*. **20**, 107–113 (2017).’

Reference [97] originally incorrectly read ‘Hlatky, M. A. The social value of childhood vaccination in the United States. *JAMA Cardiol.* **2,** 534–535 (2017).’ The corrected version of this reference is ‘Hlatky, M. A. Are novel anticoagulants worth their cost? *JAMA Cardiol*. **2**, 534–535 (2017).’

Reference [104] originally incorrectly read ‘Whiteford, H. A. et al. Interpreting scores on the Kessler psychological distress scale (K10). *Aust. NZ J. Publ. Health* **25,** 494–497 (2001).’ The corrected version of this reference is ‘Whiteford, H. A. et al. Estimating treatment rates for mental disorders in Australia. *Austr. Health Rev*. **38**, 80–85 (2014).’

Reference [115] originally incorrectly read ‘Smyth, R. et al. Tourism and natural world heritage: a complicated relationship. *Pop. Environ.* **32,** 353–375 (2011).’ The corrected version of this reference is ‘Smyth, R. et al. A study of the impact of environmental surroundings on personal well-being in urban China using a multi-item well-being indicator. *Popul. Environ.* **32**, 353–375 (2011).’ This has now been corrected in both the PDF and HTML versions of the Article.

